# Effects of the Mental Health First Aid for the suicidal person course on beliefs about suicide, stigmatising attitudes, confidence to help, and intended and actual helping actions: an evaluation

**DOI:** 10.1186/s13033-021-00459-x

**Published:** 2021-04-20

**Authors:** Kathy S. Bond, Fairlie A. Cottrill, Andrew Mackinnon, Amy J. Morgan, Claire M. Kelly, Greg Armstrong, Betty A. Kitchener, Nicola J. Reavley, Anthony F. Jorm

**Affiliations:** 1 Mental Health First Aid Australia, Parkville, VIC Australia; 2 Centre for Mental Health, Melbourne School of Population and Global Health, The University of Melbourne, Parkville, VIC Australia; 3 Nossal Institute for Global Health, Melbourne School of Population and Global Health, The University of Melbourne, Parkville, VIC Australia; 4 Kitchener Consulting, Melbourne, VIC Australia

**Keywords:** Suicide prevention, Suicidal thoughts and behaviours, Mental Health First Aid, Gatekeeper training, Public interventions

## Abstract

**Background:**

Suicide is a significant concern in Australia and globally. There is a strong argument for training community gatekeepers in how to recognise and support suicidal people in their social network. One such training course is the Mental Health First Aid for the Suicidal Person course. This course was developed using suicide prevention best practice guidelines based on expert opinion (determined using the Delphi Method).

**Methods:**

We evaluated the impact of attending the Mental Health First Aid for the Suicidal Person course on suicide literacy and stigma, confidence in and quality of intended and actual helping behaviours towards a person who is suicidal, and course satisfaction. Surveys were administered before and immediately after the course, and at 6-month follow-up. Data were analysed to yield descriptive statistics (percentages, means, standard deviations), with linear mixed models and generalized linear mixed models being used to test the statistical significance of changes over occasions of measurement.

**Results:**

We recruited 284 participants from workplaces and general community networks. The mean age was 41 years and 74% were female. 85% of people undertook the course as part of professional development, and almost half (44%) did the course because they had contact with a suicidal person. The majority (59%) of participants had previous mental health and suicide prevention training. The majority of participants held knowledge (suicide literacy) before undertaking the course. The major effect of training was to strengthen this knowledge. There was a significant improvement from pre-course (M = 1.79, SD 0.56) to post-course (M = 1.48, SD 0.82, p < 0.0001), which was maintained at follow-up (M = 1.51, SD 0.49, p < 0.0001). Confidence in gatekeeper skills significantly improved after the course and at follow-up (M = 3.15, SD 0.95 before the course to M = 4.02, SD 0.68 afterward and 3.87, SD 0.77 at follow-up, p < 0.0001 and p < 0.0001, respectively). The quality of intended helping behaviours significantly improved from pre-course (intended actions M = 4.28, SD 0.58) and to post-course (M = 4.70, SD 0.50, p < 0.0001) and were maintained at follow-up (M = 4.64, SD 0.41, p < 0.0001). There was significant improvement in some of the actions taken by participants to help a suicidal person from pre-course to post-course (e.g. asking about suicidal thoughts and plan, contacting emergency services). The course was highly acceptable to participants.

**Conclusion:**

These results indicate that this course is an acceptable intervention that delivers a broad spectrum of beneficial outcomes to community and workplace gatekeepers.

**Supplementary Information:**

The online version contains supplementary material available at 10.1186/s13033-021-00459-x.

## Background

In 2018, 3046 Australians died by suicide and it is estimated that over 65,000 Australians attempt suicide each year [1, 2]. Suicides are preventable and the Australian government has committed to reducing suicide by focusing on integrated service delivery, effective suicide prevention, coordinated support for people with severe mental illness, improved services for Indigenous people and reducing stigma and discrimination [3].

One component of an effective suicide prevention strategy involves focusing preventive efforts on individuals assessed as being at high risk of suicide by mental health and primary care services. However, recent meta-analyses have shown that individual risk assessment alone is not a good predictor of suicide [4–7]. Focusing on only individuals deemed to be at high risk ignores the larger number of people who are deemed to be at moderate and low risk and has had little effect on reducing the suicide rate [8].

While health care services have an important role in detecting and acting to reduce suicide risk, many people at risk of suicide are not in contact with these services. Less than half of people who die by suicide are in contact with primary care services in the month before their death, and only a fifth with specialist mental health care [9]. Furthermore, stigma about suicide can prevent someone from seeking professional help [10].

A complementary strategy to a focus on high-risk individuals by health professionals is a population-oriented approach. Rose’s Theorem [11] suggests that suicide events in the large numbers of ‘low-risk’ individuals may account for more cases of suicide than those among ‘high risk’ individuals. Yip and colleagues demonstrated, using a mathematical model, that addressing suicide risk factors at a population level may be more effective than targeting high-risk individuals. They suggest that both strategies are needed [8].

One population-based strategy involves public education courses that teach members of the public to recognise when someone is experiencing a mental health problem or suicidal thoughts and provide them with appropriate support (known as mental health first aid). There is evidence that people are more likely to seek help if someone in their social network (e.g. friends, family, colleagues) suggests it [12]. However, recognising suicidality can be difficult. About one-third to a half of people who die by suicide explicitly communicate their intent to family members, in other cases the indicators of suicidal intent may be unclear or misread [13]. Research has also shown that the public’s ability to act effectively to prevent suicide is lacking [14–16]. In these Australian national surveys, respondents were given a vignette of a person with depression and suicidal thoughts and asked what they would do if the person was someone they knew and cared about. Participant responses showed that while many would listen to the person, provide support and encourage professional help-seeking, very few would take the critical step of asking directly about suicidal thoughts and plans. Furthermore, people in the person’s social network may respond in a dismissive or disapproving way to the expressions of suicidal feelings, thereby shutting down communication [17, 18]. Therefore, public education on how to support a suicidal person is important.

Mental Health First Aid (MHFA) Australia develops and evaluates such education courses that have been found to be associated with higher quality support towards a suicidal person [19, 20]. The suite of MHFA Australia training includes courses that teach adults to assist other adults [21], young people [22] and Aboriginal and Torres Strait Islander people [23]. MHFA Australia has developed courses for teens assisting their peers [24, 25] and specialised courses for gambling harm [26], non-suicidal self-injury [27] and suicide [28] (the subject of this evaluation). It is important to note that MHFA Australia courses do not teach individuals to assess level of risk of suicide, but to take all talk of suicide seriously and encourage the person to get help, allowing people at all levels of risk to receive support.

In 2016, MHFA Australia launched the 4-h, specialised Mental Health First Aid for the Suicidal Person course (now called *Conversations About Suicide*).

This study employed a pre-course/post-course and 6-month follow-up design to gather data on the characteristics of the people who attended the course and to measure changes in suicide literacy and stigma, confidence in and quality of intended and actual helping behaviours towards a person who is suicidal. It also gathered information about participant satisfaction with the course.

## Methods

### Intervention

*Mental Health First Aid for the Suicidal Person* is a 4-h course that teaches participants the skills and knowledge required to provide appropriate support to a suicidal person. The training is designed to complement the 12-h Standard MHFA course and the 14-h Youth MHFA course by providing more detailed guidance on mental health first aid for suicidal thoughts. Courses are delivered by Instructors who are trained and accredited by MHFA Australia. It is based on suicide prevention best practice guidelines developed through the expert consensus of suicide consumer advocates and suicide prevention professionals [29]. The expert consensus was determined using the Delphi research method [30]. The course materials include PowerPoint slides, videos, interactive group and individual activities, and a handbook. The handbook [28] includes facts on suicide in Australia, details on how to implement the three key actions when assisting a suicidal person, and helpful resources. The course teaches three key actions that should be taken when assisting a person who is suicidal:

1. If you think someone is suicidal, ask them directly;
2. Work together to keep them safe for now;
3. Connect them to professional help.

### Procedures

Participants were recruited from Mental Health First Aid for the Suicidal Person courses that were conducted in Australia between 2017 and 2018. A research officer contacted MHFA Instructors for permission to collect evaluation data from courses they were delivering in Australian capital cities. The Instructors delivered the course in workplaces and community settings. Seven courses were restricted to employees in a workplace (support services, police and educators), 2 courses were attended by members of the public only and 15 courses were attended by a mix of employees and members of the public.

At the beginning of these courses, a research officer invited participants to enrol in the study and complete evaluation questionnaires. Participants who agreed to participate in the study were emailed a link to the follow-up survey, 6 months after the course. The follow-up survey was hosted by SurveyMonkey. Participants who had not completed the survey received three email reminders and one phone call reminder.

### Measures

The pre-course survey gathered demographic information, information about previous mental health training and personal and professional experience of suicidal thoughts and behaviours. All three surveys (pre, post and follow-up) assessed suicide literacy and stigma, confidence in and quality of intended and actual helping behaviours towards a person who is suicidal, and course satisfaction.

#### Suicide literacy

Participants were presented with five inaccurate beliefs that are addressed in the course and form part of the curriculum [31–33]:

- You should never ask a person if they are thinking about suicide, because it will put the idea in their head.
- All people who are suicidal want to die.
- Threats of suicide made when under the influence of alcohol or other drugs do not need to be taken seriously.
- If a person is talking about killing themselves then there is nothing you can do to stop them.
- You can tell how serious someone is about suicide by the method they are thinking of using.

Beliefs were rated on a 5-point Likert scale from “Strongly disagree” to “Strongly agree”. These items were included in the surveys at all three time points. A scale of beliefs was formed by averaging responses to the five items (Cronbach’s alpha = 0.71).

#### Stigma

On each occasion of measurement, participants were presented with a vignette [34] about a man named John who is experiencing suicidal thoughts. Participants were asked to imagine John is someone they know and care about and to what extent they agreed with nine statements designed to measure stigmatising attitudes about ‘people like John’. Responses were made on a 5-point Likert scale ranging from ‘Strongly disagree’ to ‘Strongly agree’. Disagreement corresponded to lower stigma. These stigma items are based on the Depression Stigma Scale [35] and form two scales, based on previous validation using exploratory structural equation modelling [36]. These measure belief that a person with a mental health problem is ‘weak not sick’, and that they are ‘dangerous or unpredictable’. These scales had Cronbach alphas of 0.82 and 0.74, respectively.

#### Confidence in and quality of intended helping behaviours

Participants were asked how confident they are in their ability to help John on a 5-point Likert scale from “Not at all” to “Extremely”. They were also asked how likely (using a 5-point Likert scale) they would be to take 12 actions—9 that are consistent with suicide prevention best practice and three that are contrary [30].

Changes in each action were examined individually. In addition, separate scales of consistent and contrary actions were created as they could show different patterns of change. A 9-item scale of recommended actions had a Cronbach’s alpha of 0.88. However, Cronbach’s alpha was only 0.35 for a scale comprising the three actions contrary to suicide prevention best practice, so these items were only examined individually.

#### Confidence in and quality of actual helping behaviours actions

Participants were asked if they had talked with anyone in the past 6 months whom they were concerned may be having suicidal thoughts. If they had, they were asked a series of questions including demographics of the person, relationship to the person and what they did to help the person. Participants were asked what actions, from a list of 13 actions they took to help the person. This list was similar to that presented with the John vignette, however, responses were binary—‘Yes/No’. Eleven actions were consistent with the suicide prevention best practice [30], while two were not. The potential actions presented were:

- Got someone else to speak to them (contrary).
- Spent time listening to the person discuss their feelings.
- Asked the person directly about suicidal thoughts.
- Asked the person directly if they had a suicide plan.
- Asked the person directly if they had attempted suicide in past.
- Allowed the person to discuss their reasons for dying and for living.
- Asked the person how they would like to be supported.
- Asked the person if there was a mental health professional whom they trusted, and sought permission to contact them on their behalf.
- Encouraged the person to get professional help as soon as possible.
- Sought their permission to contact their regular doctor or mental health professional about your concerns.
- Contacted emergency services or a mental health crisis service.
- Promised them that you would not tell anyone about their suicidal thoughts (contrary).
- Tried to convince them that suicide is wrong (contrary).
- Did you do anything else? Please specify:

They were also asked how confident they felt when actually helping the person using a 5-point Likert scale. These items were included in the pre-course and follow-up surveys.

#### Course satisfaction

In the post-course survey, participants were asked to rate their satisfaction with the course using a 5-point Likert scale including how new, how understandable and how relevant the information was, how well it was presented and their satisfaction with the course materials.

### Analysis

Mean changes on scales and, where appropriate, individual items between measurement occasions were assessed using linear mixed model repeated measures ANOVA (MMRM) with an unstructured variance–covariance matrix. Where scales were formed from multiple items, missing responses were imputed as mean values when a respondent had answered at least 75% of the items on the scale. Planned comparisons comparing measurement occasions (e.g. pre- vs. post-course) were undertaken to address specific hypotheses where appropriate. Degrees of freedom were estimated using the Kenward–Rogers method [37]. In contrast to simpler procedures such as t-tests or ANOVA which use only complete cases, mixed models retain all available data and yield an intention-to-treat estimate of change under the assumption of missingness at random.

Many of the variables evaluated had skewed distributions that were likely to yield skewed residuals, violating the model assumptions. Transforming scores was judged unlikely to be successful in dealing with these problems. Accordingly, this problem was addressed using bootstrapping and calculation of bias-corrected parameter confidence intervals to assess the robustness of conclusions reached using conventional methods. This approach was used in preference to generalised (non-linear) modelling, as it yields parameters that can be easily interpreted in terms of mean change rather than likelihood of responding in higher categories, as would be the case for ordinal or count data models.

Effect sizes were calculated using Glass’s delta [38]. This variation of Cohen’s d statistic used the pre-course standard deviation as the basis of standardisation. Values of delta reported can be interpreted as the extent of mean change induced by the course within the context of the original distribution of the variable concerned. Cohen’s definition of effect size suggests that *d* = 0.2 be considered a ‘small’ effect size, 0.5 represents a ‘medium’ effect size and 0.8 a ‘large’ effect size [39].

For the analysis of repeated binary responses (actual mental health first aid actions), a mixed effect Poisson model was used. This allows inclusion of participants who reported assisting others either before the course, at follow-up or both occasions. Unlike a logistic model, the parameters yielded reflect the relative likelihood of taking an action (rather than the odds ratio). This is appropriate when the events of interest are prevalent. Robust standard errors were used, as is appropriate when the ‘count’ data are only binary [40]. Analyses were undertaken using Stata 14.2.

## Results

### Participants

The researcher officers attended 24 courses, taught by 19 Instructors. Of the 308 course attendees approached, 284 participants were recruited. Of these, 269 (94.7%) provided at least some data immediately after the course, while a much smaller number (98, 34.5%) provided at least some responses at the 6-month follow-up. Participants who provided at least some responses at follow-up (n = 98) were compared to those who did not (n = 186) on a number of demographic attributes. Those who responded were more likely to be female (81.0% vs. 69.8%, χ^2^(1) = 4.09, p = 0.0432). Comparisons of pre-course suicide literacy, stigma, and confidence in and quality of intended helping behaviours were not statistically significant nor did they approach significance. Similarly, the proportion of each group who reported pre-course experience in responding to suicidality did not differ between groups, nor did actions taken.

Table 1 shows the demographic details of the participants. The mean age was 40.9 years and nearly three quarters were female. 86% had completed secondary school and had additional formal post-secondary qualifications.

**Table 1:** Demographics

Age—M (SD)	40.9 (13.1)	Education—N (%)	
Gender—N (%)		Year 9 or lower	3 (1.1%)
Male	74 (26.3%)	Completed year 10	14 (5.0%)
Female	204 (73.6%)	Completed year 12	22 (7.9%)
Other	0 (0%)	Trade certificate/apprenticeship	10 (3.6%)
Previous mental health training—N (%)		Other certificate	48 (17.2%)
Had previous training	168 (59.2%)	Associate or undergrad diploma	58 (20.8%)
Included suicide training	141 (83.9%)	Bachelor’s degree or higher	121 (3.4%)
MHFA course	104 (61.9%)	Other	3 (1.4%)

The overwhelming majority of participants (85%) undertook the course for workplace or professional reasons, while nearly half (44%) reported having had contact with suicidal people in the past, as a reason for participation. Over half of the participants (59%) had previous mental health training and, for the majority of these participants, this training included guidance in assisting suicidal people. Of participants who had previous training, 62% had previously taken a Mental Health First Aid (MHFA) Australia course.

Most participants (79.8%) reported having some experience of suicide outside their professional roles. This included with family, friends and their wider community. Notably, nearly 1 in 5 (18%) participants reported having experienced suicidality themselves.

### Suicide literacy

Most participants held beliefs consistent with course teaching before the training. Between 84.5 and 94.0% of participants responded either ‘Disagree’ or ‘Strongly Disagree’ to the first four of the five inaccurate beliefs before the course. In contrast, only 57.2% of participants disagreed or strongly disagreed to the last item (“You can tell how serious someone is about suicide by the method they are thinking of using”).

Mean item responses on each occasion of measurement are shown in Table 2. The statistical significance of changes in mean responses was tested using linear mixed models. All changes from pre-course values to both post-course and follow-up values were statistically significant. Changes from post-course to follow-up were not generally significant. Effect sizes ranged from small to medium size effects. See Additional file 1 for the distribution of responses to each of these statements at each time point.

**Table 2:** Means and standard deviations for belief

Item	Pre-course		Post-course			6-month Follow-up		
	Mean	SD	Mean	SD	∆	Mean	SD	∆
You should not ask a person if they are thinking about suicide	1.76	0.86	1.28	0.82	0.56	1.27	0.58	0.58
All people who are suicidal want to die	1.78	0.73	1.47	0.80	0.42	1.51	0.63	0.36
Suicidal threats made when under the influence of alcohol or other drugs, do not need to be taken seriously	1.49	0.69	1.33	0.82	0.24	1.32	0.67	0.25
If a person is talking about killing themselves there is nothing you can do	1.51	0.70	1.41	0.74	0.15	1.30	0.61	0.31
You can tell how serious someone is about suicide by the method they are thinking of using	2.42	1.11	1.93	1.25	0.45	2.14	1.22	0.25
Scale (average)	1.79	0.56	1.48	0.67	0.56	1.51	0.49	0.51

Glass’s Delta compared to pre-course mean using pre-course standard deviation; number of observations varies slightly due to missing data

Changes in means on the overall belief scale showed the same pattern as for individual items, with significant improvements post-course, which were maintained at follow-up (t(267.0) = 7.48, p < 0.0001; and t(133.6) = 5.12, p < 0.0001, respectively). These changes were of medium effect size (see Table 2).

### Intended helping behaviours

#### Recommended actions

Before the course, most participants responded that they would be ‘Likely’ or ‘Very likely’ to take each of the recommended actions. Percentages generally ranged from 70% (asking about suicide and previous attempts) to over 96% (listening). Asking about a plan was an outlier, with only 56.8% of likely or very likely responses. Despite the high rates of concordance, there were substantial increases in the proportion of participants responding ‘Very likely’ rather than ‘Likely’ after the course, e.g. listening or asking about support. Accordingly, analysis of change in mean responses using MMRMs found statistically significant changes for all actions, both after the course and at follow-up (see Table 3). Effect sizes were largest for asking about a plan, asking directly about suicide, and asking about previous attempts. Some actions showed a small, but significant, increase in means from after the course to follow-up. These included asking about suicide, asking about a plan, and allowing John to discuss why he wants to die.

**Table 3:** Action items consistent with best practice guidelines—vignette

Item	Pre-course		Post-course			6-month follow-up		
	Mean	SD	Mean	SD	∆	Mean	SD	∆
*Spend time listening to John talk about his feelings*	4.56	0.58	4.74	0.57	0.32	4.74	0.46	0.31
Ask John directly about suicidal thoughts	3.96	0.92	4.69	0.62	0.79	4.50	0.71	0.58
*Ask John directly if he has a plan for how he will kill himself*	3.66	1.14	4.62	0.67	0.85	4.31	0.84	0.57
*Ask John directly if he has attempted suicide in past*	3.89	1.02	4.57	0.66	0.67	4.43	0.74	0.53
*Allow John to discuss his reasons for wanting to die*	4.23	0.81	4.67	0.56	0.54	4.61	0.55	0.47
*Ask John how he would like to be supported*	4.55	0.63	4.75	0.54	0.32	4.79	0.43	0.38
*Provide John with information about where he can get help*	4.60	0.64	4.80	0.52	0.31	4.81	0.42	0.33
*Ask John if there is a mental health professional whom he trusts, and seek permission to contact them on his behalf*	4.45	0.79	4.72	0.57	0.34	4.70	0.51	0.31
*Encourage John to get professional help as soon as possible*	4.64	0.60	4.76	0.52	0.20	4.85	0.38	0.35
Scale (average)*	4.28	0.58	4.70	0.50	0.72	4.64	0.41	0.61

*p < 0.0001

Change on the overall scale of recommended actions reflected the pattern of individual action items. The increase in likelihood of taking recommended actions was significant after the course and maintained at follow-up (t(282.6) = 10.12, p < 0.0001; and t(185.4) = 6.49, p < 0.0001 respectively). The slight decrease in means from post course to follow-up was also significant (t(106.1) = 3.29, p = 0.0013). The changes from before the course were of medium to large size (see Table 3).

**Fig. 1:**
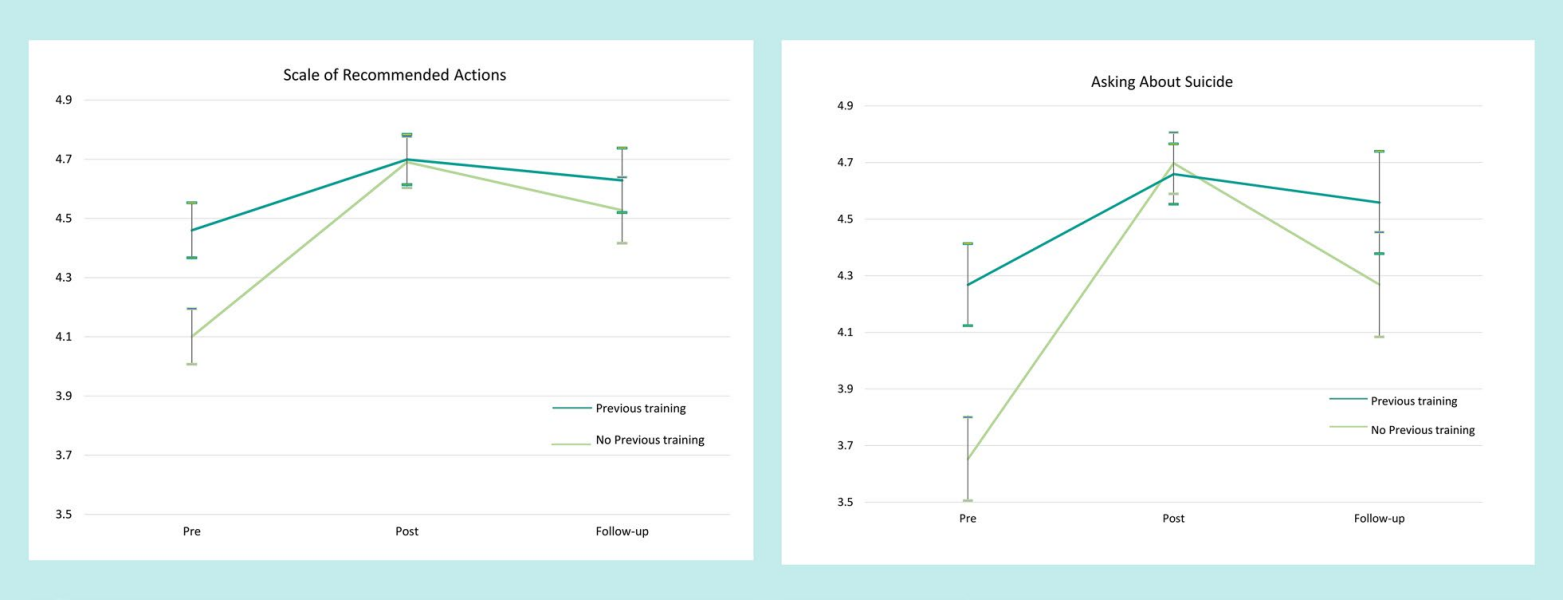
Action items consistent with suicide prevention best practice comparing those with and without previous training

Further analysis showed that those with no previous training that included guidance in assisting suicidal people were less likely to consider taking the actions consistent with the course before the course than those with previous training (t(282.1) = 5.33, p < 0.0001). After the course, both groups improved, with those with no previous training scoring similarly to those with training (t(262.9) = 0.14, p = 0.8863; and t(135.2) = 1.26, p = 0.2110 after the course and at follow-up respectively). Similar results were found with the specific action of asking the person directly about suicidal thoughts (see Fig. 1.)

#### Contrary actions

Of the three contrary action items (those not recommended), only ‘Wait and see’ showed a substantial increase in the proportion of participants responding ‘Very unlikely’ rather than ‘Unlikely’ after the course. Before the course, over 80% of participants indicated they would be ‘Unlikely’ or ‘Very unlikely’ to take this action, split almost equally between these alternatives. After the course, the proportion of participants responding ‘Very unlikely’ alone approached 80%. Unsupported responses (‘Not sure’ to ‘Very likely’) substantially declined (12.4% to 2.3%).

‘Promising not to tell’ showed a similar but substantially attenuated overall pattern. Before the course, just over half the participants (54.4%) indicated they would be ‘Unlikely’ or ‘Very unlikely’ not to promise to tell anyone. After the course, 51.7% responded ‘Very unlikely.’ However, there remained considerable non-supported responses (21%), which was maintained at follow-up (20.8%).

Responses to the action item of ‘Try to convince John that suicide is wrong’ were quite different to other actions. Before the course, more participants reported that they would be more likely than unlikely to do this. After the course, over half the participants (56.3%) moved to a supported position (‘Unlikely’ or ‘Very unlikely’), but nearly a third (31.6%) gave unsupported responses (‘Likely’ or ‘Very likely’). This proportion increased to over 40% at follow-up.

Analysis of change in mean responses to each action item using mixed models found statistically significant reductions for all items, both after the course and at follow-up (see Table 4 for means). Some action items showed a small, but significant increase in means from after the course to follow-up. There was a substantial reduction in the overall scale of contraindicated actions after the course and at follow-up (t(263.8) = 13.03, p < 0.0001; and t(113.2) = 4.52, p < 0.0001 respectively). The rebounds from post course to follow-up was also significant (t(108.8) = 3.73, p = 0.0003). The changes from before the course were of medium to large size (see Table 3). Consistent with the recommended actions scale, those with previous training in assisting suicidal people were less likely to consider taking non recommended actions before the course (t(279.3) = 4.31, p < 0.0001). This difference was maintained after the course and at follow-up despite the improvements attributable to the course in both groups.

**Table 4:** Actions not consistent with best practice guidelines—vignette

Item	Pre-course		Post-course			6-month follow-up		
	Mean	SD	Mean	SD	∆	Mean	SD	∆
*Wait and see if things get worse before speaking to John*	1.72	0.80	1.24	0.57	− 0.60	1.38	0.65	− 0.43
*Try to convince John that suicide is wrong*	3.15	1.16	2.58	1.49	− 0.49	3.00	1.44	− 0.13
*Promise John that you will not tell anyone about his suicidal thoughts*	2.57	1.31	1.90	1.23	− 0.51	1.95	1.16	− 0.48

p < 0.0001 for (average of scores)

#### Confidence in ability to help John

Confidence to assist ‘John,’ moved from a modal response of ‘Moderately’ [confident] (40.0%) to ‘Quite a bit’ [confident] after the course (62.4%) and at follow-up (55.2%). The proportion of participants who felt ‘Not at all’ [confident] or ‘Only a little bit’ [confident] fell from a quarter (25.0%) to just 2.4% after the course. Consistent with this, mean responses increased from 3.15 (SD 0.95) before the course to 4.02 (SD 0.68) afterward and 3.87 (SD 0.77) at follow-up, corresponding to large effects sizes (∆ = 0.91 and ∆ = 0.76, respectively). Change from pre-course means at both times was statistically significant (t(276.9) = 15.07, p < 0.0001; and t(126.6) = 10.35, p < 0.0001, respectively). The slight decline in confidence from after the course to follow-up approached statistical significance (t(121.7) = 1.94, p = 0.0541). The pattern of confidence as a function of previous training followed that seen for contrary actions: those with previous training had higher mean scores before the course (t(276.0) = 3.77, p = 0.0002) and essentially maintained this difference at both occasions afterwards.

### Stigma

‘Weak not sick’ score distributions were highly skewed, with 40.4% of participants having the minimum possible score, indicating favourable attitudes. Nevertheless, there were reductions in scores after the course and at follow-up (t(275.6) = 8.89, p < 0.0001; and t(132.7) = 2.66, p < 0.01, respectively). The slight increase in the mean from post-course to follow-up was also significant (t(106.1) = 2.75, p < 0.01). The changes from pre-course were small to medium size (see Table 5).

**Table 5:** Stigma scales

Item	Pre-course		Post-course			6-month follow-up		
	Mean	SD	Mean	SD	∆	Mean	SD	∆
‘Weak not sick’	6.02	2.44	4.99	1.84	0.43	5.22	1.95	0.33
‘Dangerous/unpredictable’	9.99	2.81	8.28	2.65	0.61	8.72	2.72	0.46

The ‘Dangerous/Unpredictable’ score distributions were much less skewed, with a modal response of 8 (just below an average ‘Disagree’ response). Changes in means from pre-course were significant both post-course and at follow-up (t(267.0) = 11.74, p < 0.0001; and t(125.5) = 3.81, p = 0.0002, respectively). The increase in the mean from post-course to follow-up was also significant (t(101.4) = 2.68, p = 0.0071). The changes from pre-course were medium to large in size (see Table 5).

### Actions taken to help a suicidal person

Nearly two thirds of participants (66.2%)^1^ reported assisting a suicidal person within the 6 months prior to the course. Most participants who had assisted, reported helping just one (43.6%) or a small number of people (2 or 3, 30.2%), but a sizable proportion reported assisting four or more people (26.3% before the course and 17.3% after). Individuals assisted were most often clients or patients (47.4% of those assisted), but were sometimes friends or immediate family members (17.7% and 17.1%, respectively). A slightly lower percentage (58.9%) reported assisting someone at follow-up. Just over a quarter of participants (27.8%) who reported assisting at 6-month follow-up did not report having done so prior to the course.

**Fig. 2:**
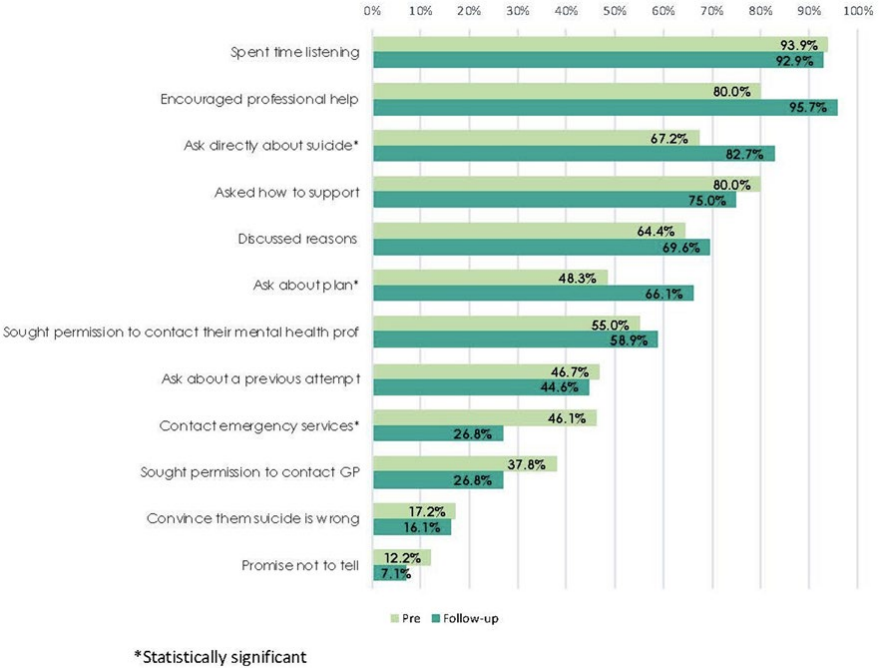
Percentage of participants taking particular actions

Figure 2 shows the percentage of participants taking each of the presented actions pre-course and at follow-up. Overall, the pattern of actions was very similar at both time points. Nearly all those who assisted reported spending time listening to the person and encouraging them to get professional help as quickly as possible. They were also less likely to report trying to convince the person that suicide is wrong and promising not tell anyone else (contraindicated actions). There was a significant increase in asking directly about suicide and about a plan to act on their suicidal thoughts. There were significant decreases in involving other people in helping, with decreases in contacting emergency services and getting another person to speak to the individual being statistically significant (see Table 6). This may indicate that participants are less likely to take panic-oriented actions.

**Table 6:** Relative likelihood (RL) of taking particular actions when assisting a suicidal person

Action	RL	95% CI	z	p-value
Got someone else to speak to them (contrary)	0.63	0.40–0.99	− 2.01	0.044
Spent time listening to the person discuss their feelings	0.99	0.91–1.07	− 0.28	0.780
Asked the person directly about suicidal thoughts	1.22	1.05–1.43	2.51	0.012
Asked the person directly if they had a suicide plan	1.37	1.08–1.73	2.59	0.010
Asked the person directly if they had attempted suicide in past	0.96	0.70–1.31	− 0.27	0.787
Allowed the person to discuss their reasons for dying and for living	1.08	0.89–1.32	0.77	0.441
Asked the person how they would like to be supported	0.94	0.80–1.10	− 0.78	0.435
Asked the person if there was a mental health professional whom they trusted, and sought permission to contact them on their behalf	1.07	0.83–1.38	0.53	0.596
Encouraged the person to get professional help as soon as possible	1.07	0.94–1.22	1.05	0.294
Sought their permission to contact their regular doctor or mental health professional about your concerns	0.71	0.45–1.13	− 1.46	0.144
Contacted emergency services or a mental health crisis service	0.58	0.38–0.90	− 2.44	0.015
Promised them that you would not tell anyone about their suicidal thoughts (contrary)	0.58	0.22–1.54	− 1.09	0.276
Tried to convince them that suicide is wrong (contrary)	0.93	0.48–1.81	− 0.21	0.834

### Satisfaction with the course

Almost 95% of participants stated that the course was well presented and relevant to them. Satisfaction for the course materials (i.e. the handbook, slides, videos, and activities) was similarly high (89%).

**Fig. 3:**
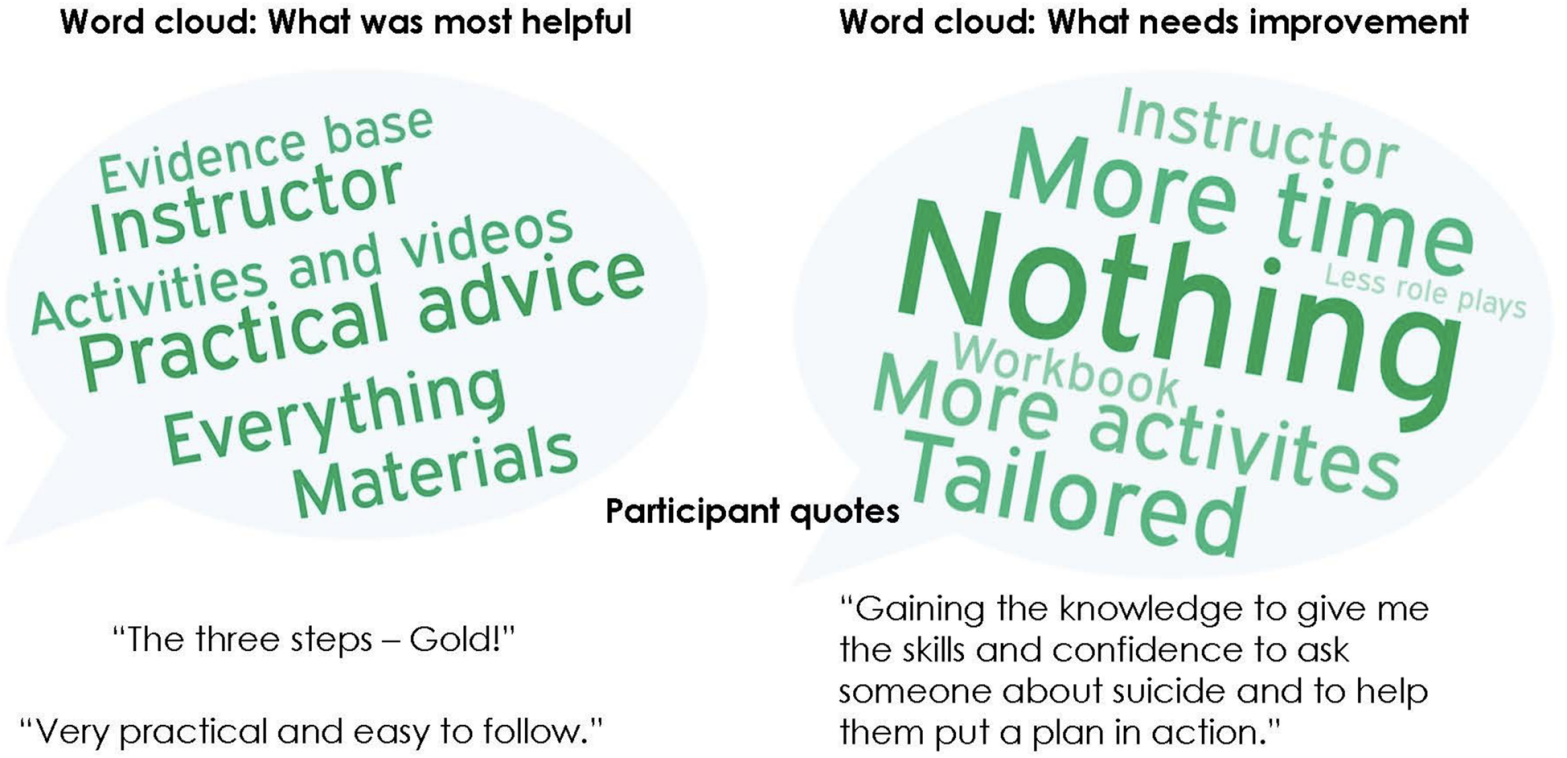
Summary of qualitative data

Qualitative data (see Fig. 3 for a summary) suggests that the participants thought the course was practical, and in particular, participants found the three key actions helpful. The course activities were also well regarded, including group discussions, role plays and videos that depict someone providing mental health first aid to a suicidal person. Participants also identified areas for improvement including the desire to have a longer course and the inclusion of more activities (role plays, videos, scenarios and group discussions).

## Discussion

This evaluation of the Mental Health First Aid for the Suicidal Person course included pre-course, post-course and 6-month follow-up design to gather data on the characteristics of the people who attended the course and to measure changes in suicide literacy and stigma, confidence in and quality of intended and actual helping behaviours towards a person who is suicidal. It also gathered information about participant satisfaction with the course. Overall, the results of this study are similar to evaluations of other mental health first aid courses where the course was found to increase knowledge and confidence, decrease stigma and improve the quality of intentions to help a suicidal person [19, 41, 42]. The evidence that the course positively influences actual helping actions is less clear due to low response rates at follow-up. However, an evaluation of a similar course for Australian Aboriginal and Torres Strait Islander people observed substantial gains in both intended and actual helping actions [42].

Many participants in this study had previous training and a range of experiences of suicide. Accordingly, many of their beliefs about how to help a suicidal person were at the most informed end of possible responses. The course served as a refresher for these participants, as there were clear and statistically significant improvements for them after the course. An evaluation of another gatekeeper training program found similar results in participants with and without previous training [43]. The responses of the participants with no previous training improved to levels similar to those with previous training. These improvements were statistically significant at post-course and at follow-up and were of medium effect size. While the changes were maintained at follow-up for both groups, they were stronger for those with prior training, indicating that regular gatekeeper training (i.e. refresher training) may be helpful in maintaining skills.

Helping a suicidal person was a common experience for participants both at pre-course and follow-up. The types of assistance given were similar over occasions, but after attending the course, participants reported less involvement of others, including contacting emergency services. There were notable increases in explicit questioning about suicide. Taken together, these changes suggest greater confidence to connect and engage with the person by listening and providing interpersonal support rather than too quickly calling emergency services. Of note, the responses to the action item about trying to convince the person that suicide is wrong were quite different to other actions, with there being virtually no change from pre to follow-up scores. This action is contrary to suicide prevention best practice (the course teaches that you should not try to convince the person that suicide is wrong). It is quite natural to want to convince someone that suicide is wrong, as the consequences of suicide are so grave. A greater emphasis on this may be needed in future iterations of the course.

The aim of the Mental Health First Aid for the Suicidal Person course is to teach skills that enable participants to support a suicidal person in a way that reduces the chance that the person will act on suicidal thoughts. The results of this study suggest that the course promotes positive and helpful actions, although limited by missing data. However, the results are strengthened when considered within the context of other research. Studies have found that mental health first aid intentions predict mental health first aid actions. A national Australian study [20] that surveyed 3002 people, asked participants about their intentions to assist a suicidal person in a vignette and about any actions they had taken to assist a suicidal person. The study found that best practice intentions were highly correlated with best practice actions. In another study [44], 2005 young people were interviewed 2 years after being presented with vignettes and asked about their intentions to help the person in the vignette. At follow-up, 608 reported assisting a person with a mental health problem and first aid intentions and beliefs about the helpfulness of certain first aid actions predicted the actions that they actually took. Finally, 820 Australians were surveyed 6 months apart and were asked about intentions toward a person in a vignette and if they had assisted someone they knew. People who intended to assess and assist for mental health crises (e.g. ask about thoughts of suicide) were five times more likely to do this with someone they knew at follow-up. The quality of past intentions and behaviours, as well as the person’s confidence in the ability to help, were the most significant predictors of supportive behaviour at follow-up [45].

The second way to infer appropriate actions towards a suicidal person based on intentions is to map the results of this evaluation to behavioural change models. The Theory of Planned Behaviour [46] suggests that changing beliefs is the first step in a chain of events that leads to a change in behaviour. The beliefs include beliefs about the consequences of the behaviour (behavioural beliefs), beliefs about what they think others think (normative beliefs), and beliefs about factors that may hinder or enhance the success of the behaviour (control beliefs or self-efficacy). In the context of this study, behavioural beliefs include the belief that asking about suicide will not put the idea in their head and that you can make a life-saving difference to a suicidal person, and control beliefs include increased confidence in one’s ability to assist a suicidal person. These beliefs lead to the formation of behavioural intentions, which as reviewed above, are a good indicator of future behaviour. A randomised controlled trial would further strengthen the evidence that the course promotes positive and helpful actions.

### Limitations

There are a few limitations to this evaluation. First, there was no control group. Second, the low response rate at follow-up made it difficult to draw firm conclusions about whether the course improved actual first aid actions taken to support a suicidal person. However, intentions improved and other studies have found intentions to be a good indicator of behaviour. Another limitation is the high level of knowledge held by participants prior to taking the course. Future research needs to investigate how well these results generalise to other populations that might be less knowledgeable. The bias towards female participants was striking. These limitations may say something about the dissemination of the course and some attention to this is warranted to ensure the course is available to the people who need it most.

## Conclusion

Many people who die by suicide explicitly communicate their intent to family members [13]. Furthermore, evidence suggests that people are more likely to seek help if someone in their social network suggest it [12]. Therefore, it is important to train community gatekeepers how to recognise and support a person who is experiencing suicidal thoughts. The results of this study indicate that those who undertake this course may be able to provide life-saving help. Further research is needed to explore the impact of the course on supportive behaviours towards people who are experiencing suicidal thoughts and a randomised controlled trial would further strengthen the evidence for this course.

## Supplementary Information

**Additional file 1. **Distribution of responses to the beliefs about suicide items.

**Additional file 2. **SPSS data file.

## Data Availability

See Additional file 2.

## Abbreviation

MHFA: Mental Health First Aid

## Footnotes

1 One participant did not report helping one or more people, but reported taking action to help a suicidal person and so is included as having assisted.

## References

[1] Australia Bureau of Statistics. Causes of death, Australia 2018. Contract No. 3303.0. Canberra; 2019.

[2] Black Dog Institute. Facts about suicide in Australia. 2018. https://www.blackdoginstitute.org.au/resources-support/suicide-self-harm/facts-about-suicide-in-australia/. Accessed 18 June 2020.

[3] Australian Government Department of Health. The fifth national mental health and suicide prevention plan. Canberra; 2017.

[4] McHugh CM, Corderoy A, Ryan CJ, Hickie IB, Large MM (2019). Association between suicidal ideation and suicide: meta-analyses of odds ratios, sensitivity, specificity and positive predictive value. BJPsych Open.

[5] Carter G, Milner A, McGill K, Pirkis J, Kapur N, Spittal M (2017). Predicting suicidal behaviour using clinical instrucments: systematic review and meta-analysis of positive predictive values for risk scales. Br J Psychiatry.

[6] Chan M, Bhatti H, Meader N, Stockton S, Evans J, O’Connor R (2016). Predicting suicide following self-harm: systematic review of risk factors and risk. Br J Psychiatry.

[7] Large M, Kaneson M, Myles N, Myles H, Gunaratne P, Ryan C (2016). Meta-analysis of longitudinal cohort studies of suicide risk assessment among psychiatric patients: heterogeneity in results and lack of improvement over time. PLoS ONE.

[8] Yip PSF, So BK, Kawachi I, Zhang Y (2014). A Markov chain model for studying suicide dynamics: an illustration of the Rose theorem. BMC Public Health.

[9] Stene-Larsen K, Reneflot A (2019). Contact with primary and mental health care prior to suicide: a systematic review of the literature from 2000 to 2017. Scand J Public Health.

[10] Barney LJ, Griffiths KM, Jorm AF, Christensen H (2006). Stigma about depression and its impact on help-seeking intentions. Aust New Zeal J Psychiatry.

[11] Sher L (2019). Is it time to employ Rose’s theorem to prevent suicide?. Aust N Z J Psychiatry.

[12] Vogel DL, Wade NG, Wester SR, Larson L, Hackler AH (2007). Seeking help from a mental health professional: the influence of one’s social network. J Clin Psychol.

[13] Isometsä ET (2001). Psychological autopsy studies—a review. Eur Psychiatry.

[14] Rossetto A, Jorm AF, Reavley NJ (2014). Quality of helping behaviours of members of the public towards a person with a mental illness: a descriptive analysis of data from an Australian national survey. Ann Gen Psychiatry.

[15] Nicholas A, Pirkis J, Jorm A, Spittal MJ, Reavley N (2019). Helping actions given and received in response to suicide risk: findings from an Australian nationally representative telephone survey. SSM Popul Health.

[16] Nicholas A, Pirkis J, Rossetto A, Jorm A, Spittal M, Reavley N (2020). Confidence and intentions to help a person at risk of suicide. Suicide Life Threat Behav.

[17] Owens C, Owen G, Belam J, Lloyd K, Rapport F, Donovan J (2011). Recognising and responding to suicidal crisis within family and social networks: qualitative study. BMJ.

[18] Sweeney L, Owens C, Malone K (2015). Communication and interpretation of emotional distress within the friendships of young Irish men prior to suicide: a qualitative study. Health Soc Care Community.

[19] Morgan AJ, Ross A, Reavley NJ (2018). Systematic review and meta-analysis of mental health first aid training: effects on knowledge, stigma, and helping behaviour. PLoS ONE.

[20] Jorm AF, Nicholas A, Pirkis J, Rossetto A, Reavley NJ (2018). Associations of training to assist a suicidal person with subsequent quality of support: results from a national survey of the Australian public. BMC Psychiatry.

[21] Kitchener BA, Jorm AF, Kelly CM (2017). Mental Health First Aid Manual.

[22] Kelly C, Kitchener B, Jorm A (2017). Youth mental health first aid manual.

[23] Bin Talib S, Bond K, Kelly C (2017). Aboriginal and Torres Strait Islander mental health first aid manual.

[24] Kelly C, Hart L, Kitchener B, Jorm A (2016). Teen mental health frist aid: a manual for young people in years 7–9 helping their friends.

[25] Hart L, Kelly C, Kitchener B, Jorm A (2015). Teen mental health first aid: a manual for young people in years 10–12 helping their friends.

[26] Kelly L (2018). Conversations about gambling: course handbook.

[27] Kelly L (2018). Coversations about non-suicidal self-injury: course handbook.

[28] Kelly C, Blee F, Caessen G (2016). Conversations about suicide: course handbook.

[29] Mental Health First Aid (2014). Suicidal thoughts and behaviours: MHFA guidelines (revised 2014).

[30] Ross AM, Kelly CM, Jorm AF (2014). Re-development of mental health first aid guidelines for suicidal ideation and behaviour: a Delphi study. BMC Psychiatry.

[31] Samaritans. Myths about suicide. https://www.samaritans.org/how-we-can-help/if-youre-worried-about-someone-else/myths-about-suicide/. Accessed 18 June 2020.

[32] Caruso K. Suicide myths. http://www.suicide.org/suicide-myths.html. Accessed 18 June 2020.

[33] Relationships Australia. Suicide: facts and myths. https://www.square.org.au/introduction/suicide-facts-and-myths/. Accessed 18 June 2020.

[34] Jorm AF, Blewitt KA, Griffiths KM, Kitchener BA, Parslow RA (2005). Mental health first aid responses of the public: results from an Australian national survey. BMC Psychiatry.

[35] Griffiths KM, Christensen H, Jorm AF, Evans K, Groves C (2004). Effect of web-based depression literacy and cognitive–behavioural therapy interventions on stigmatising attitudes to depression: randomised controlled trial. Br J Psychiatry.

[36] Yap MBH, Mackinnon A, Reavley N, Jorm AF (2014). The measurement properties of stigmatizing attitudes towards mental disorders: results from two community surveys. Int J Methods Psychiatr Res.

[37] Kenward MG, Roger JH (1997). Small sample inference for fixed effects from restricted maximum likelihood. Biometrics.

[38] Smith ML, Glass GV (1977). Meta-analysis of psychotherapy outcome studies. American psychologist.

[39] Cohen J (1992). Quantitative methods in psychology: a power primer. Psychol Bull.

[40] Zou G (2004). A modified poisson regression approach to prospective studies with binary data. Am J Epidemiol.

[41] Hadlaczky G, Hökby S, Mkrtchian A, Carli V, Wasserman D (2014). Mental health first aid is an effective public health intervention for improving knowledge, attitudes, and behaviour: a meta-analysis. Int Rev Psychiatry.

[42] Armstrong G, Sutherland G, Pross E, Mackinnon A, Reavley N, Jorm AF (2020). Talking about suicide: an uncontrolled trial of the effects of an Aboriginal and Torres Strait Islander mental health first aid program on knowledge, attitudes and intended and actual assisting actions. PLoS ONE.

[43] Haggerty D, Carlson JS, McNall M, Lee KS, Williams S (2019). Exploring youth mental health first aider training outcomes by workforce affiliation: a survey of Project AWARE participants. School Ment Health.

[44] Yap MBH, Jorm AF (2012). Young people’s mental health first aid intentions and beliefs prospectively predict their actions: findings from an Australian national survey of youth. Psychiatry Res.

[45] Rossetto A, Jorm AF, Reavley NJ (2016). Predictors of adults’ helping intentions and behaviours towards a person with a mental illness: a six-month follow-up study. Psychiatry Res.

[46] Ajzen I (2002). Perceived behavioral control, self-efficacy, locus of control, and the theory of planned behavior. J Appl Soc Psychol.

