# Supplementary material for: Effects of the Mental Health First Aid for the suicidal person course on beliefs about suicide, stigmatising attitudes, confidence to help, and intended and actual helping actions: an evaluation
Source: Int J Ment Health Syst. 2021 Apr 20;15:36. doi: 10.1186/s13033-021-00459-x (PMC8056520; doi:10.1186/s13033-021-00459-x)

# Additional File 1: Distribution of responses to the beliefs about suicide items

You should never ask a person if they are thinking about suicide, because it will put the idea in their head.

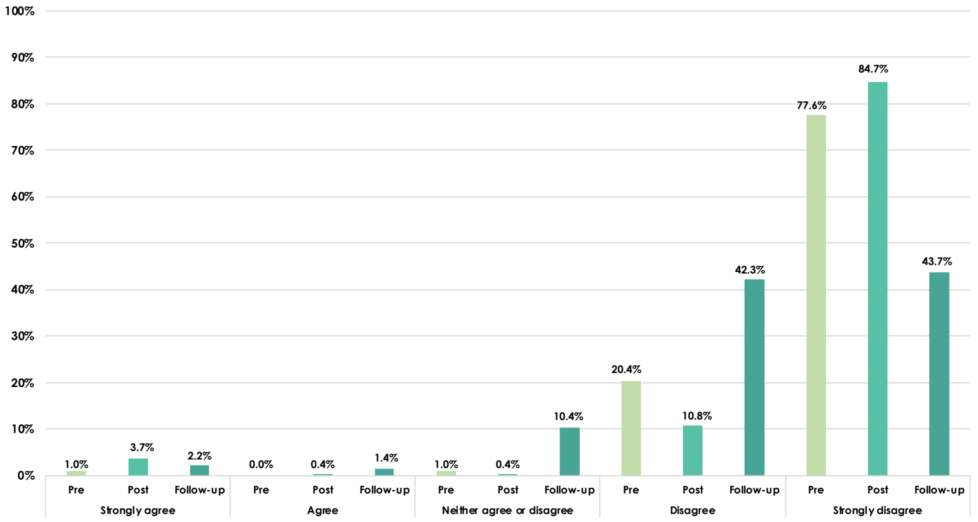


All people who are suicidal want to die.

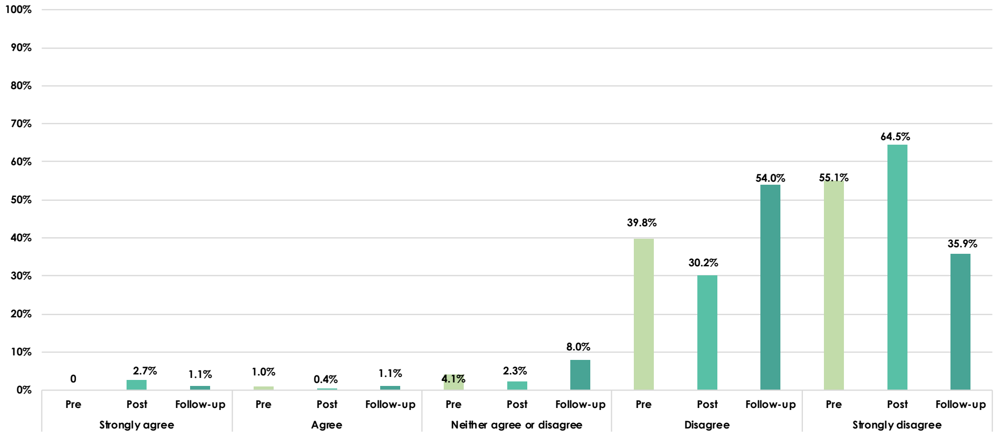

If a person is talking about killing themselves then there is nothing you can do to stop a suicidal person.

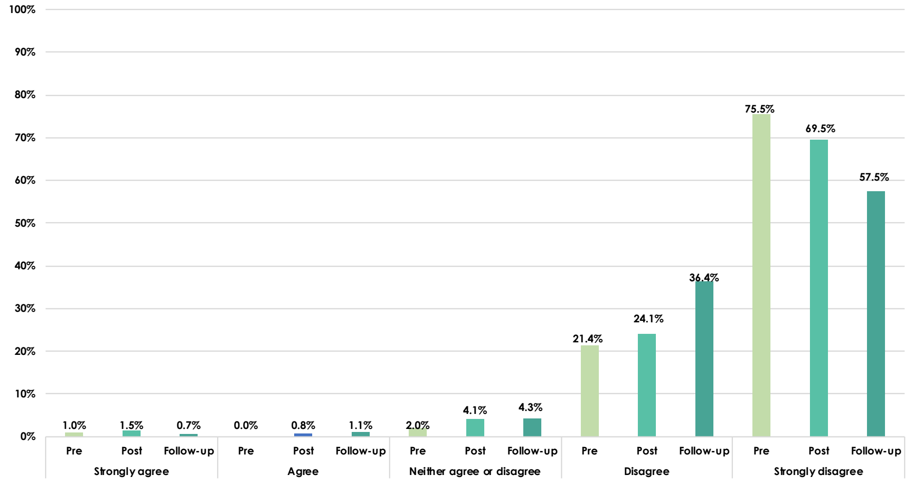


Suicidal threats made while under the influence of alcohol or other drugs do not need to be taken seriously.

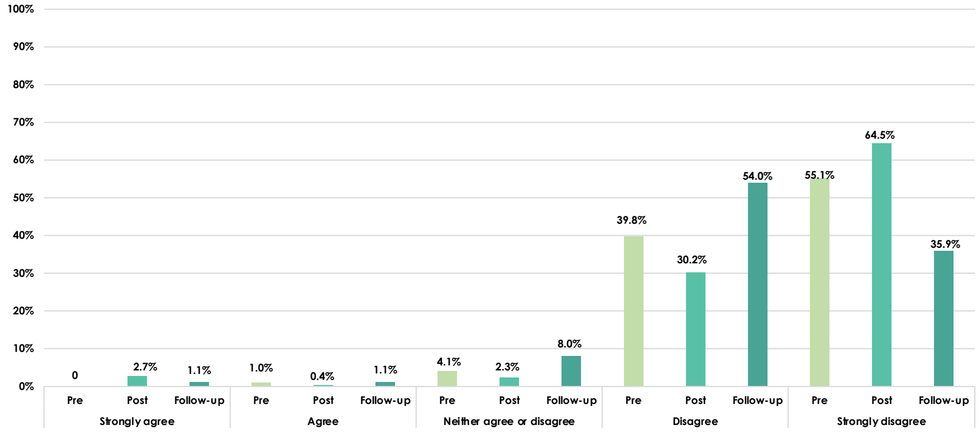

You can tell how serious someone is about suicide by the method they are thinking about using.

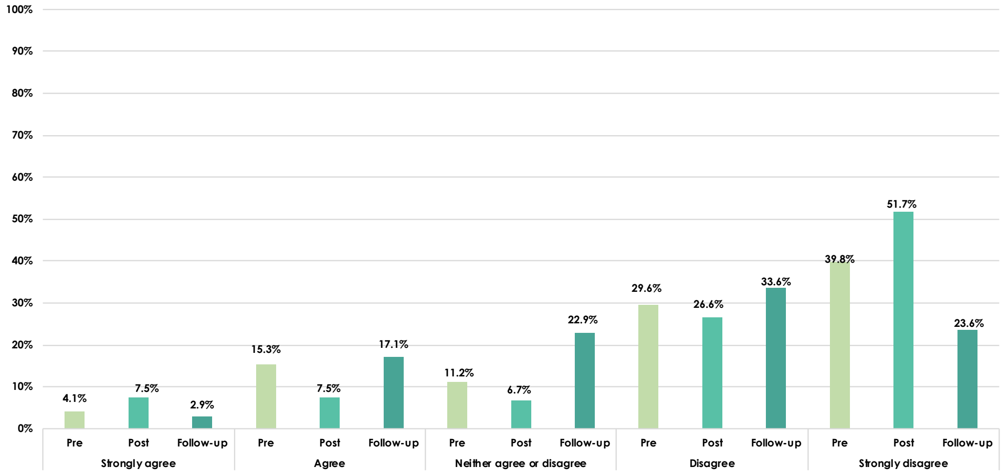

Supplement: Supplementary file 1 — Additional file 1. Distribution of responses to the beliefs about suicide items. [file 13033_2021_459_MOESM1_ESM.pdf]
